# Supplementary material for: Boldness, activity, and aggression: Insights from a large-scale study in Baltic salmon (Salmo salar L)
Source: PLoS One. 2023 Jul 20;18(7):e0287836. doi: 10.1371/journal.pone.0287836 (PMC10358900; doi:10.1371/journal.pone.0287836)
Supplement: Attachment — Submitted filename: Respons to reviewers comments.docx [file pone.0287836.s002.docx]

Reviewers' comments:

Reviewer's Responses to Questions

**Comments to the Author**

1. If the authors have adequately addressed your comments raised in a previous round of review and you feel that this manuscript is now acceptable for publication, you may indicate that here to bypass the “Comments to the Author” section, enter your conflict of interest statement in the “Confidential to Editor” section, and submit your "Accept" recommendation.

Reviewer #1: All comments have been addressed

Reviewer #2: All comments have been addressed

Reviewer #3: All comments have been addressed

2. Is the manuscript technically sound, and do the data support the conclusions?

The manuscript must describe a technically sound piece of scientific research with data that supports the conclusions. Experiments must have been conducted rigorously, with appropriate controls, replication, and sample sizes. The conclusions must be drawn appropriately based on the data presented.

Reviewer #1: Yes

Reviewer #2: Yes

Reviewer #3: Yes

3. Has the statistical analysis been performed appropriately and rigorously?

Reviewer #1: Yes

Reviewer #2: Yes

Reviewer #3: Yes

4. Have the authors made all data underlying the findings in their manuscript fully available?

The [PLOS Data policy](http://www.plosone.org/static/policies.action#sharing) requires authors to make all data underlying the findings described in their manuscript fully available without restriction, with rare exception (please refer to the Data Availability Statement in the manuscript PDF file). The data should be provided as part of the manuscript or its supporting information, or deposited to a public repository. For example, in addition to summary statistics, the data points behind means, medians and variance measures should be available. If there are restrictions on publicly sharing data—e.g. participant privacy or use of data from a third party—those must be specified.

Reviewer #1: Yes

Reviewer #2: No

Raw data has been included in the submission of the revised version.

Reviewer #3: Yes

5. Is the manuscript presented in an intelligible fashion and written in standard English?

PLOS ONE does not copyedit accepted manuscripts, so the language in submitted articles must be clear, correct, and unambiguous. Any typographical or grammatical errors should be corrected at revision, so please note any specific errors here.

Reviewer #1: Yes

Reviewer #2: Yes

Reviewer #3: Yes

6. Review Comments to the Author

Please use the space provided to explain your answers to the questions above. You may also include additional comments for the author, including concerns about dual publication, research ethics, or publication ethics. (Please upload your review as an attachment if it exceeds 20,000 characters)

Reviewer #1: The authors answer my concerns and they carefully revised the manuscript following my comments and those from other reviewers. I feel that the manuscript has been greatly improved and is ready for publication. I only have two minor comments regarding the revised version, please see below:

L61-64: there is a mix of concept between personalities and coping styles in these lines (which is not the case in the following lines where it is clearer). So, please clarify the lines 61-64.

The text has been changed to: “It has becoming increasingly clear that salmonids and other teleosts display intraspecific divergence in behavioral and physiological responses to challenge [23]. Behavioral  responses is often described as personalities, which include boldness, exploration, activity, aggression, and sociability [24]. Physiological responses are often called stress coping and includes physiological traits, such as sympathetic reactivity and post-stress plasma cortisol [25].” (line 62-66)

L503-509: I overall agree with the authors statements regarding the difficulties for measuring consistency of behavioural response in aquaculture and the causes of such difficulty (life stages, size, environmental parameters). Please add some reference to support the statements.

Thank you for your comment. In order to support our statements, the following references was added: Stamps and Grothius (2010)

Reviewer #2: The authors have done a great work addressing most points raised and the manuscript. However, in the version I received the figures were in a very bad quality and it was impossible to check that the requested changes on the figures have been made. In addition, here below a few specific comments.

We are sorry about the bad quality of the figures you received. However, the figures submitted submission portal were in high resolution and when checking submitted files we found that they were of good quality and clearly readable. However, we fully agree that in the manuscript the quality is very bad. Thus, something is happening during the submission process which is not in our hands to control.

L219: Why temperature was removed from this model?
Reply: we decided to not include temperature after extensive visual exploration of the data. In contrast to locomotory variables distance moved in arena, proportion of time moving in arena and distance moved in mirror zone, there was no clear effect of temperature on the proportion of time spent in the center zone or mirror zone. The effect of temperature on locomotory behavior of fish is also well established in the literature [54](line 241-243).
Thank you for clarifying the reason why temperature was excluded from those models. I would only suggest to remove the sentence in line 242: “Morover the effect of temperature on locomotory behaviour of fish is also well established in literature [54]” because not relevant to the previous statement. The use of space, such as center zone and mirror zone, is not necessarily strictly related to locomotion behaviour, in addition, if there was an effect of temperature on locomotion behaviour then temperature should be included and finally, the the reference seems to be about a study that has been done on growth and not on locomotion behaviour.

“Thank you for your comment, the line 242 has now been removed”

Line 111: Not clear what response to boldness at group level means. In addition, I think this paragraph should be discussed and integrated in the previous paragraph when presenting and discussing the objectives of the study.

“Thank you for your comment. Boldness on a group level refers to a test where the individual results is not tracked but the results is taken into an account for the whole group. In order to clarify the above statement. In an attempt to make this more clear the text ahs been changed: “Behavioral tests at group level, e.g. response to hypoxia, boldness, have been described for several teleosts. However, when testing fish in groups behavioral responses may be confound by agonistic interactions. Moreover, in salmon there is limited information on how responses to hypoxia and/or boldness determined in groups relates to aggressive behavior of individual fish.” (line 108-111).

Reviewer #3: The authors have addressed my comments appropriately and the manuscript has improved. I just have two more queries.

Line 157 – 158: Provide trial numbers along with total sample size.

In total we performed 137 trials. This information has now has been added (line 164).

Line 241-243: I’m needing more assurance that temperature did not have a confounding effect on boldness. An ~11 ˚C temperature differentiation over the experiment is significant and therefore potential temperature effects need to be substantially checked and accounted for. I see you mention to another reviewer that the effects were "visually assessed" however it wouldn't be too time-consuming to compare the explanatory of models with and without temperature. I suggest this is done. I also advice you provide reviewers with the raw data so that we can help with tasks like these.

We found no effect of temperature on time spent in the center zone or mirror zone and the text has been changed to:“…although here we did not include a random effect of temperature since there was no clear effect of temperature on the proportion of time spent in the center zone (r2=0.0035) or mirror zone (r2=0.0017). In both cases we used pooled within-test-minute correlations. Despite those values were significant (p<0.001), we regarded the level of significance as inflated due to multiple comparison, and in addition, the effect size were regarded as to low to be relevant.” (line 240-245).

7. PLOS authors have the option to publish the peer review history of their article ([what does this mean?](https://journals.plos.org/plosone/s/editorial-and-peer-review-process#loc-peer-review-history)). If published, this will include your full peer review and any attached files.


If you choose “no”, your identity will remain anonymous but your review may still be made public.


**Do you want your identity to be public for this peer review?** For information about this choice, including consent withdrawal, please see our [Privacy Policy](https://www.plos.org/privacy-policy).

Reviewer #1: No

Reviewer #2: No

Reviewer #3: No
